# A novel method of detecting raised intracranial pressure from head computed tomography using optic nerve sheath diameter

**DOI:** 10.1186/2197-425X-3-S1-A816

**Published:** 2015-10-01

**Authors:** MG Povey, FA Amey, CR Bassford

**Affiliations:** Warwick Medical School, Warwick University, Coventry, United Kingdom; Critical Care Unit, University Hospital Coventry, Coventry, United Kingdom

## Introduction

Raised intracranial pressure (ICP) can cause secondary brain injury, which is associated with severe disability and mortality [1]. Invasive ICP monitoring has been linked to increased mortality [2]. Sekhon, et al. [3] demonstrated a strong correlation between optic nerve sheath diameter (ONSD) on CT scan and ICP, with the potential to use this non-invasive method to detect raised ICP.

## Objectives

To assess the efficacy of ONSD as a predictor of raised ICP and to determine whether predictive value can be improved by controlling for variables measurable on CT.

## Methods

Single centre, retrospective study of patients receiving ICP monitoring during 2013. For each patient, the following measurements were recorded (***A****,****B*** and ***L*** recorded bilaterally):

- ONSD 3mm behind the globe - maximum recorded (***A***)

- ONSD half way between the globe and the superior orbital fissure (SOF) - average recorded (***B***)

- Distance from the globe to the SOF (***L***)

- Anterior-posterior diameter of the foramen magnum (***FM***)

Optic nerve ratio (ONR) and ValX were calculated using equations 1 and 2, respectively (Figyre 1). The strength of the relationship between ValX and ICP was assessed using Pearson´s correlation coefficient (r). A receiver operating characteristic (ROC) curve was produced to assess the ability of ValX to predict ICP above 15mmHg. A subset was re-measured by a second assessor and interclass correlation coefficient (ICC) was used to assess inter-rater reliability.

**Figure 1:**
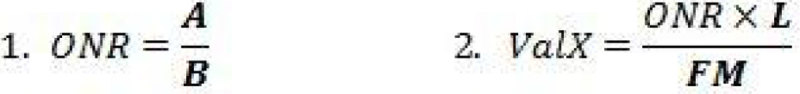
**Calculation of ONR and ValX.**

**Figure 2:**
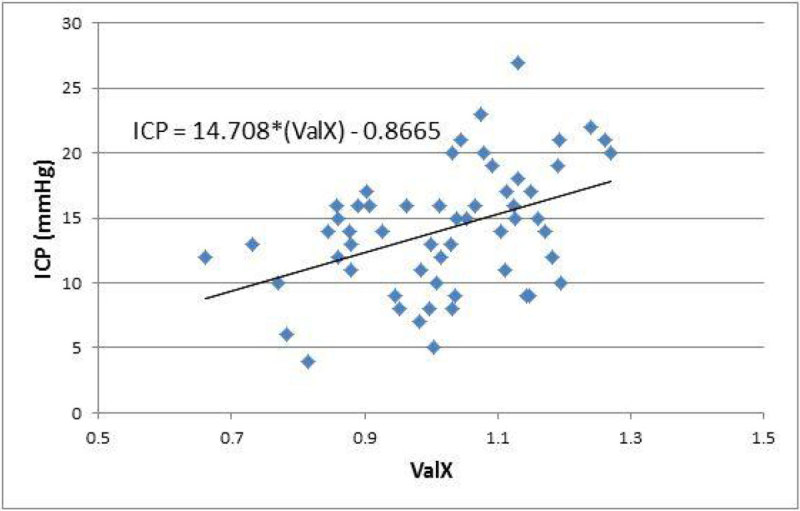
**[Scatter graph of ICP against ValX].**

**Figure 3:**
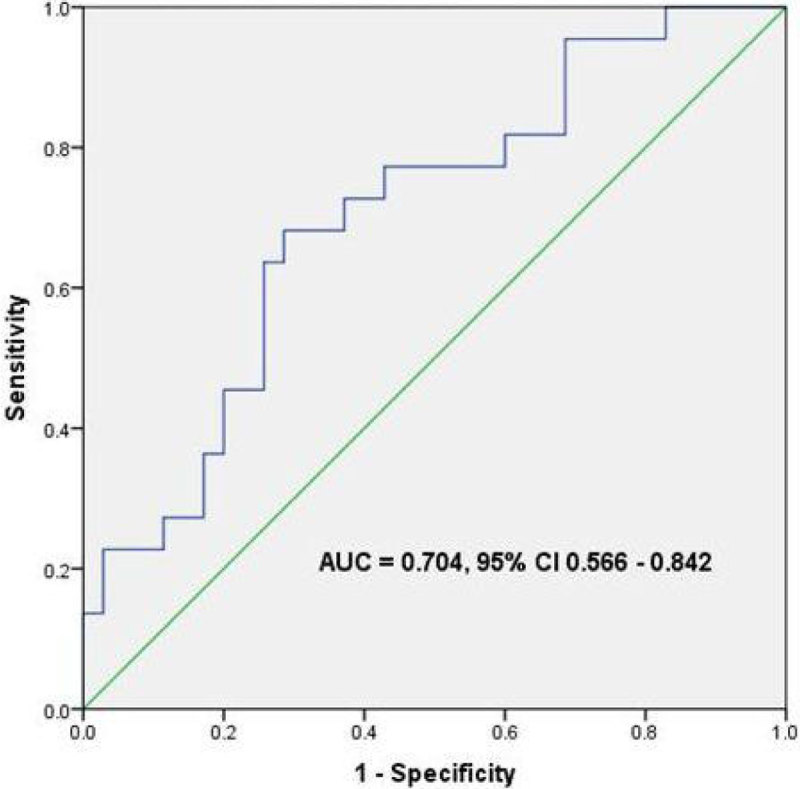
**[ROC curve: Ability of ValX to identify ICP>15mmHg].**

## Results

57 head CTs were identified where simultaneously recorded ICP was available. The mean value of ValX was 1.02 (SD = 0.138) and mean ICP was 14.1mmHg (SD = 4.8mmHg). No correlation was identified between ICP and ONSD (r = 0.032, n = 57). There was a moderate correlation between ValX and ICP (r = 0.427, p = 0.001). The ICC was 0.98 (95% CI 0.96 to 0.99). ValX had an area under the curve to discriminate elevated ICP (>15 mmHg) of 0.70 (95% CI 0.57 to 0.84). Using a cut-off of 1.03, ValX had a sensitivity of 73%, specificity of 63%, positive predictive value of 55% and a negative predictive value of 79%.

## Conclusions

We were unable to replicate the relationship observed by Sekhon et al. [3] between ICP and ONSD. However, by controlling for measurements ***L*** and ***FM***, we found a moderately strong relationship. This novel technique has good inter-rater reliability. More work is required to develop this method of excluding raised ICP.
